# Author’s Response Regarding Cardiac Disease Patterns in Northern Malawi: Epidemiologic Transition Perspective

**DOI:** 10.2188/jea.JE20090104

**Published:** 2009-09-05

**Authors:** Elsayed Z Soliman, Hadge Juma

**Affiliations:** 1 Epidemiological Cardiology Research Center (EPICARE), Department of Epidemiology and Prevention, Wake Forest University School of Medicine, Winston-Salem, NC, USA; 2 Mzuzu Central Hospital, Mzuzu, Malawi

We thank O. S. Ogah for his interest in our article “Cardiac Disease Patterns in Northern Malawi: Epidemiologic Transition Perspective”^1^. In his letter,^2^ Ogah agrees with us that the current epidemiologic transition in Northern Malawi is characterized by new patterns of cardiovascular diseases (CVD). Ogah, however, believes that we might have been incorrect when we stated that there was no similar previous study on the population that we investigated. In support of this argument he cites two previous studies that he mistakenly thought were conducted on our study population. As is clear from the title of our article, our study population is from northern Malawi, which is totally different from the populations in the two studies he cites. The first study^3^ Ogah mentions was conducted in Zimbabwe—a totally different country! The second^4^ was a very small study that was conducted over 30 years ago in southern, not northern, Malawi. It should be noted that northern Malawi is populated by a very different ethnic group that speaks a completely different language from that found in southern Malawi. Despite his misunderstanding of the geography, we thank Ogah for highlighting these two studies, as their findings support our observations regarding the emergence of new CVD in sub-Saharan Africa. We believe that our findings from northern Malawi, in addition to other data from the region, definitely enhance our understanding of the epidemiology of CVD in sub-Saharan Africa and in developing countries.
